# Editorial: Innovative methods for sleep staging using neuroinformatics

**DOI:** 10.3389/fninf.2024.1448591

**Published:** 2024-07-08

**Authors:** Antonio Fernández-Caballero, Michel Le Van Quyen

**Affiliations:** ^1^Departamento de Sistemas Informáticos, Escuela Técnica Superior de Ingenieros Industriales, Universidad de Castilla-La Mancha, Albacete, Spain; ^2^Neurocognition and Emotion Unit, Instituto de Investigación en Informática de Albacete, Universidad de Castilla-La Mancha, Albacete, Spain; ^3^Cognition and Psychosis Group, CIBERSAM (Biomedical Research Networking Centre in Mental Health), Madrid, Spain; ^4^Laboratoire d'imagerie médicale, Institut National de la Santé et de la Recherche Médicale (INSERM), Paris, France; ^5^Cortex and Epilepsy Group, Institut du Cerveau et de la Moelle épiniére (ICM), Paris, France

**Keywords:** sleep staging, neurinformatics, sleep disorder, machine learning, deep learning

## 1 Introduction

Sleep staging and analysis play a critical role in understanding sleep mechanisms and diagnosing sleep disorders (Thorpy, 2012). Traditionally, sleep staging has required manual scoring of electroencephalography (EEG) data, which is labor-intensive and prone to error (Lee et al., 2022).

Neuroinformatics methods, such as machine learning and deep learning (Górriz et al., 2023), look to automate and standardize the sleep staging process (Faust et al., 2019). These methods have helped improve the accuracy and reliability of sleep staging by providing objective measures of sleep parameters that were previously subjective and prone to inter-observer variability. These tools have revolutionized the field of sleep research, enabling clinicians and researchers to gain deeper insights into the mechanisms of sleep and sleep-related disorders (Dai et al., 2021).

This Research Topic on “*Innovative methods for sleep staging using neuroinformatics*” aimed to explore innovative ways to stage the different phases of sleep using advanced technology and computational tools related to Neuroinformatics.

The scope of this Research Topic included, among others

- Development of open-source software tools for sleep staging and analysis using Neuroinformatics methods.
- Evaluation of novel spectral measures that can accurately detect changes in sleep stages during overnight studies.
- Investigation of deep learning models that can accurately predict sleep stage annotations and improve the accuracy of sleep-related diagnoses.
- Research on the applicability and effectiveness of innovative Neuroinformatics methods in improving the accuracy and efficiency of sleep staging, ultimately improving the diagnosis and treatment of sleep disorders.
- Comparison of the performance of Neuroinformatics methods with traditional manual sleep staging methods, including assessment of their inter-rater reliability and reproducibility.
- Investigation of the limitations and challenges of Neuroinformatics methods for sleep staging and analysis, including potential biases in machine learning models and the need for expert oversight and validation.

This Research Topic on “*Innovative methods for sleep staging using neuroinformatics*” has provided a means for researchers and academics who have a current or developing interest in the area of sleep staging and analysis by incorporating innovative Neuroinformatics methods, and ultimately to improve the accuracy and efficiency of sleep-related diagnosis and treatment. In the end, we accepted a total of seven papers for this Research Topic.

## 2 The papers

Of the seven papers, five were Original Research papers, one was a Methods paper, and the last was a Technology and Code paper.

The Technology and Code paper “*Robin's Viewer: Using deep-learning predictions to assist EEG annotation*” by Weiler et al. addresses the problem of using machine learning techniques to assist EEG annotation in automated artifact detection, sleep staging, and seizure detection. More precisely, the authors face the problem that fully automated processes do not provide the possibility to inspect the output of the models and to re-evaluate possible false predictions. Therefore, Robin's Viewer (RV), an EEG viewer for annotating time-series EEG data, is presented. The key feature that distinguishes RV from existing EEG viewers is the visualization of output predictions from deep learning models trained to detect patterns in EEG data. RV is described as an EEG viewer that combines the predictive power of deep learning models with the knowledge of scientists and clinicians to optimize EEG annotation.

The Methods paper by Schneider et al. “*Scale-free and oscillatory spectral measures of sleep stages in humans*” highlights a solution to the fact that traditional band-based spectral methods ignore the fundamental structure of EEG spectra, which consists of two main components, a decaying power law corresponding to aperiodic neural background activity, and spectral peaks present due to neural oscillations. These are therefore susceptible to misrepresenting the underlying phenomena. Therefore, the authors introduce a fitting method that attempts to separate and parameterize the aperiodic and periodic spectral components, called fitting oscillations and one over f (FOOOF), which was applied to a set of annotated whole-night sleep EEG recordings.

A first Original Research paper “*Effect of total sleep deprivation on effective EEG connectivity for young male in resting-state networks in different eye states*” by Ma et al. addresses the effect of total sleep deprivation (TSD) on resting-state functional networks, specifically the default mode network (DMN) and the sensorimotor network (SMN), using functional connectivity. According to the authors, while it is known that the activities of these networks differ depending on eye state, it is still unclear how TSD affects them in different eye states. Therefore, the aim of this paper was to investigate the effect of TSD on DMN and SMN in different eye states using effective functional connectivity via isolated effective coherence in precise low-resolution brain electromagnetic tomography.

The second Original Research paper by Li Y. et al. “*A study on feature selection using multi-domain feature extraction for automated k-complex detection*” examines k-complex detection as it plays an important role in the field of sleep research. The authors claim that it is necessary to implement automatic detection methods based on classical machine learning algorithms. However, due to the complexity of the EEG signal, current feature extraction methods always produce low relevance for k-complex detection, which leads to a large loss in detection performance. Therefore, firstly, multi-domain features based on time, spectral analysis and chaotic theory are extracted. Then, several feature selection methods are explored and their performance in k-complex detection is compared. Finally, three classical classifiers are used to evaluate the performance of the feature selection models.

The third Original Research paper by Li J. et al. “*Few-shot EEG sleep staging based on transductive prototype optimization network*” proposes a few-shot EEG sleep staging method called transductive prototype optimization network (TPON). The method aims to improve the performance of EEG sleep staging. Compared with traditional deep learning methods, TPON uses a meta-learning algorithm that generalizes the classifier to new classes that are not visible in the training set and have only a few examples for each new class. The prototypes are learned from existing objects through meta-training, and the prototype distribution of the class is optimized by using the support set and unlabeled samples with high confidence.

Another Original Research paper “*Validation of spectral sleep scoring with polysomnography using forehead EEG device*” by Onton et al. highlights a spectral scoring approach that addresses all the current shortcomings of visual scoring, including high cost, time-consuming, susceptibility to human variability, patient discomfort, lack of visualization to validate the hypnogram, and no recognition of differences between delta and slow-wave deep sleep. Previous algorithms have used spectral information to classify traditional visual stages. The proposed method uses the clearly visible spectral patterns to develop new spectral stages that are similar to, but different from, visual stages. The study compares traditional visual scoring from 32-channel polysomnography with forehead-only spectral scoring from a concurrently worn EEG patch.

The last Original Research paper by Holm et al. “*An optimized framework for processing multicentric polysomnographic data incorporating expert human oversight*” is also based on polysomnographic recordings. Their approach is devoted to the question of how exactly the algorithms using relevant features from polysomnography should be incorporated into the workflow of sleep technologists. Therefore, the paper presents a data collection platform developed within the Sleep Revolution project to utilize polysomnographic data from several European centers.

## 3 Conclusion

The seven papers together have received about 30,000 views as of June 4, 2024. This confirms the popularity of sleep staging and analysis as a topic for readers. We, the guest editors of this Research Topic, believe in the growing importance of sleep staging and analysis in fields as diverse as computer science, engineering, biology, psychology, medicine, and neuroscience. We also believe that sleep staging has not yet reached its full potential. We foresee a tremendous growth of solutions using the sleep staging/Neuroinformatics binomial in the near future.

## Author contributions

AF-C: Funding acquisition, Supervision, Validation, Writing – original draft, Writing – review & editing. ML: Supervision, Validation, Writing – original draft, Writing – review & editing.
